# Supplementary material for: CAPTURE-24: A large dataset of wrist-worn activity tracker data collected in the wild for human activity recognition
Source: Sci Data. 2024 Oct 16;11:1135. doi: 10.1038/s41597-024-03960-3 (PMC11484779; doi:10.1038/s41597-024-03960-3)
Supplement: Supplementary file 1 — Supplementary file [file 41597_2024_3960_MOESM1_ESM.pdf]

Supplementary Information

A List of hand-crafted features

The following commonly used features [1] (40 in total) are extracted from the raw accelerometry for the random forest and XGBoost models:

- **Quantiles** Minimum, maximum, median, 25th and 75th percentiles of acceleration for each of the three axis streams as well as the magnitude stream.
- **Correlations** Correlation between axes and 1-sec-lag autocorrelation of the magnitude stream.
- **Spectral features** First and second dominant frequencies and their powers, and spectral entropy.
- **Peak characteristics** Number of peaks and median prominence of the peaks.
- **Angular features** Estimated dynamic roll, pitch and yaw (mean and standard deviation), and gravity roll, pitch and yaw (mean).

B Hyperparameter tuning details

B.1 Baseline architecture

The final architecture is described in Table S1. Here, $\mathrm{Conv}(k,n)$ means a 1D convolution with n filters of kernel size k, $m \times \mathrm{ResBlock}(k,n)$ means m residual blocks of size m with n filters and kernel size k [2], $\mathrm{Drop}(p)$ is dropout [3] with rate p, $\mathrm{FC}(n)$ is a fully connected layer with output size n, $\mathrm{BiLSTM}(n)$ is a bidirectional LSTM [4] with output size n, and finally, $\mathrm{Linear}(n)$ is a linear layer with output size n. As usual, batch normalization [5] and rectified linear units [6] follow the Conv layers. Rectified linear units also follow the FC layer. All convolutions use a stride and circular padding of 1. Downsampling is performed with anti-aliasing as described in [7].

Supplementary Table 1. Network architectures for convolution neural network (CNN) and recurrent neural network (RNN)

State size	Layer
(*, 3, 1000)	Conv(3, 128) / 2
(*, 128, 500)	Conv(3, 128), 3 x ResBlock(3, 128) / 2
(*, 128, 250)	Conv(3, 256), 3 x ResBlock(3, 256) / 2
(*, 256, 125)	Conv(3, 256), 3 x ResBlock(3, 256) / 5
(*, 256, 25)	Conv(3, 512), 3 x ResBlock(3, 512) / 5
(*, 512, 5)	Conv(3, 512), 3 x ResBlock(3, 512) / 5
(*, 512, 1)	Drop(0.5), FC(1024) or BiLSTM(512 (x2))
(*, 1024)	Linear(6)

For the RNN model, BiLSTM is used in place of FC in order to ingest sequences of windows – we limit the maximum sequence length to 8.

We tried $k \in \{3,5\}$ for the kernel sizes and $m \in \{0,1,2,3\}$ for number of residual blocks (constrained to be the same throughout), an initial configuration of filters $n = 64 \to 64 \to 128 \to 128 \to 256 \to 256 \to 512$, and a wider one $n = 128 \to 128 \to 256 \to 256 \to 512 \to 512 \to 1024$. We used ASHA [8] as implemented in Ray Tune [9].

B.2 Data augmentation

We tried four data augmentation techniques [10]: jittering, time warping, magnitude warping, and shifting. For jittering, we tried standard deviation $\sigma \in \{0, .01, .05, .1\}$. For time and magnitude warping, $\sigma \in \{0, .01, .05, .1\}$ and knots $\in \{2, 4\}$. For shifting, shift $\in \{0 \text{ sec}, 1 \text{ sec}, 2 \text{ sec}, 5 \text{ sec}\}$. To reduce computational cost, each augmentation technique is tried independently and the best parameters are then combined. Each trial is run until early-stopped with patience of 5. Table S2 reports the best parameters found. Note in particular that we did not find jittering to improve performance. For the other techniques, we found slight to moderate improvements.

Supplementary Table 2. **Data augmentation parameters**

Technique	Parameters
Jittering	$\sigma = 0$
Time warping	$\sigma = .05$, knots $= 4$
Magnitude warping	$\sigma = .05$, knots $= 2$
Shifting	shift $= 2$ sec

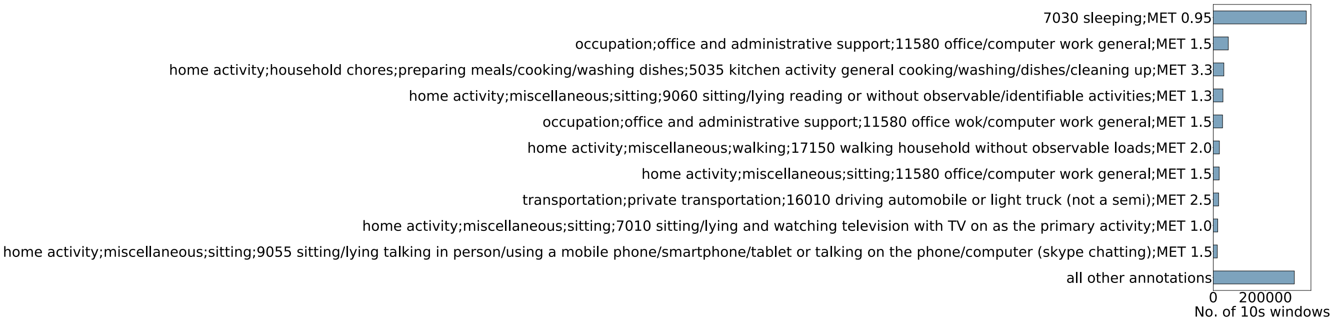

Supplementary Figure 1. **Top 10 most frequent Compendium of Physical Activities code annotations found in Capture-24**

B.3 Optimization

Initially, we tuned the architecture and data augmentation parameters using Adam [11] with learning rate $\eta = 3 \times 10^{-3}$. After tuning was done, we retrained the optimal model using stochastic gradient descent with restarts [12, 13] and tried initial learning rates $\eta \in \{.1, .15, .2, .25, .3, .35, .4, .45\}$. The trials were run until early-stopped with patience of 5. The CNN model converged in around 30 epochs while the RNN model in around 40 epochs.

B.4 Computational resources

Models were trained using a V100 GPU with 32GB of RAM. Training time varies by model and task, but can all be completed within 12h.

C Annotations

Figure S1 plots the occurrence of the 10 most common CPA code annotations found in Capture-24. The rest of the annotations are displayed as “all other annotations” in the diagram to indicate the long-tail distribution over the codes.

D Other datasets

Scores for other public datasets using the same benchmark models are shown in Table S3. As these datasets are very small, the test scores can have high variance as well as being prone to p-hacking (it is tempting to redo the train/test split several times to get a desired conclusion). We therefore perform leave-one-subject-out cross-testing. For WISDM [14] dataset (51 subjects), 10-fold cross-testing is used instead. For simplicity, we show results for RF and CNN only, each being an archetype of traditional and modern methods, respectively. Unsurprisingly, we observe that CNN underperforms in the smaller datasets (ADL [15] and PAMAP2 [16]) while RF is rather consistent across dataset sizes. On the other hand, CNN performs on par or better than RF in the larger datasets (RealWorld [17] and WISDM), as well as in CAPTURE-24 (results in the main text). We also note that the performances are overall higher than those of CAPTURE-24, which is expected as these datasets are collected in a clean lab setting.

E Distribution of Coarse Activity Labels

In Figure S2, we show the activity distribution using the 6-class and 10-class schemes[18].

Supplementary Table 3. **Scores (median and interquartile range) for other public datasets using same benchmark models.**

ADL ($n = 2.7$ hrs)

Model	F1-score	Cohen's κ	Pearson-Yule's ϕ
RF	.777 (.671, .868)	.694 (.642, .846)	.734 (.661, .857)
CNN	.604 (.568, .697)	.558 (.484, .646)	.576 (.538, .679)

PAMAP2 ($n = 4.5$ hrs)

Model	F1-score	Cohen's κ	Pearson-Yule's ϕ
RF	.810 (.762, .829)	.810 (.751, .824)	.812 (.756, .826)
CNN	.685 (.625, .708)	.696 (.619, .710)	.711 (.629, .721)

RealWorld ($n = 18$ hrs)

Model	F1-score	Cohen's κ	Pearson-Yule's ϕ
RF	.775 (.674, .857)	.732 (.620, .824)	.743 (.640, .827)
CNN	.806 (.700, .884)	.771 (.639, .871)	.781 (.654, .876)

WISDM ($n = 43$ hrs)

Model	F1-score	Cohen's κ	Pearson-Yule's ϕ
RF	.805 (.747, .824)	.752 (.689, .776)	.756 (.693, .778)
CNN	.804 (.755, .846)	.747 (.714, .804)	.752 (.727, .807)

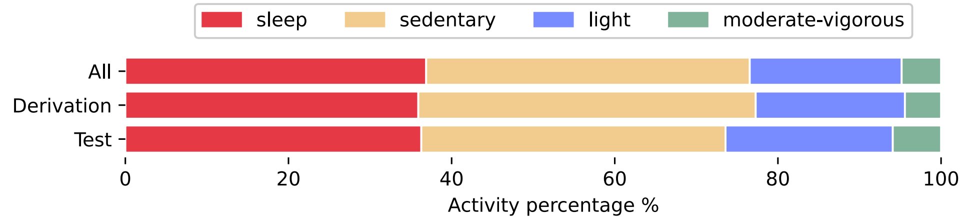

(a) Four classes

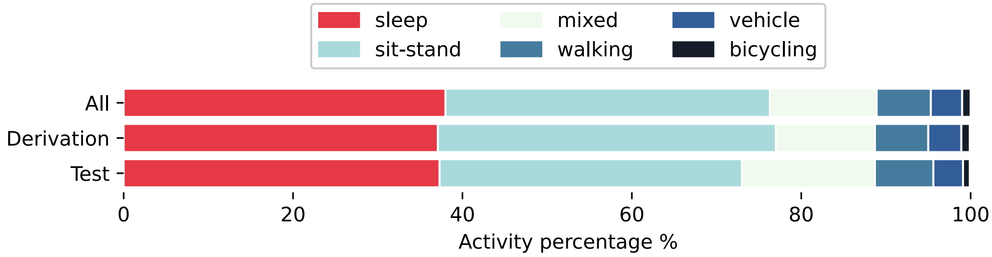

(b) Six classes

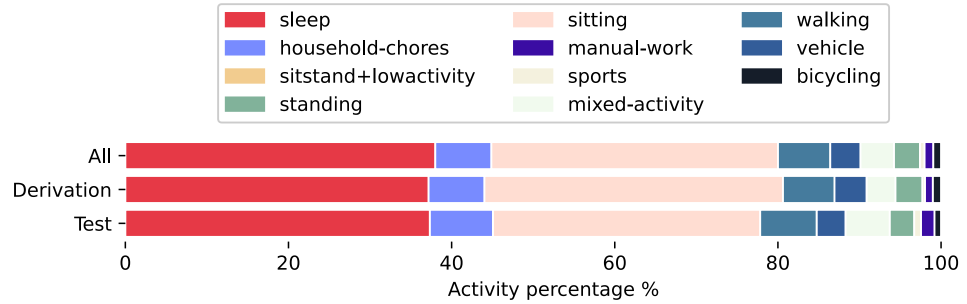

(c) Ten classes

Supplementary Figure 2. Distribution of activities different labelling schema

References

1. Twomey, N. *et al. A comprehensive study of activity recognition using accelerometers* in *Informatics* **5** (2018), 27.
2. He, K., Zhang, X., Ren, S. & Sun, J. *Identity mappings in deep residual networks* in *ECCV* (2016), 630–645.
3. Srivastava, N., Hinton, G., Krizhevsky, A., Sutskever, I. & Salakhutdinov, R. Dropout: a simple way to prevent neural networks from overfitting. *The journal of machine learning research* **15,** 1929–1958 (2014).
4. Hochreiter, S. & Schmidhuber, J. Long short-term memory. *Neural computation* **9,** 1735–1780 (1997).
5. Ioffe, S. & Szegedy, C. *Batch normalization: Accelerating deep network training by reducing internal covariate shift* in *International conference on machine learning* (2015), 448–456.
6. Fukushima, K. & Miyake, S. in *Competition and cooperation in neural nets* 267–285 (Springer, 1982).
7. Zhang, R. *Making convolutional networks shift-invariant again* in *International conference on machine learning* (2019), 7324–7334.
8. Li, L. *et al.* A system for massively parallel hyperparameter tuning. *arXiv preprint arXiv:1810.05934* (2018).
9. Liaw, R. *et al.* Tune: A research platform for distributed model selection and training. *arXiv preprint arXiv:1807.05118* (2018).
10. Um, T. T. *et al. Data augmentation of wearable sensor data for parkinson's disease monitoring using convolutional neural networks* in *Proceedings of the 19th ACM International Conference on Multimodal Interaction* (2017), 216–220.
11. Kingma, D. P. & Ba, J. Adam: A method for stochastic optimization. *arXiv preprint arXiv:1412.6980* (2014).
12. Loshchilov, I. & Hutter, F. Sgdr: Stochastic gradient descent with warm restarts. *arXiv preprint arXiv:1608.03983* (2016).
13. Smith, L. N. *Cyclical learning rates for training neural networks* in *2017 IEEE winter conference on applications of computer vision (WACV)* (2017), 464–472.
14. Weiss, G. M., Yoneda, K. & Hayajneh, T. Smartphone and smartwatch-based biometrics using activities of daily living. *IEEE Access* **7,** 133190–133202 (2019).
15. Bruno, B., Mastrogiovanni, F., Sgorbissa, A., Vernazza, T. & Zaccaria, R. *Analysis of human behavior recognition algorithms based on acceleration data* in *2013 IEEE International Conference on Robotics and Automation* (2013), 1602–1607.
16. Reiss, A. & Stricker, D. *Introducing a new benchmarked dataset for activity monitoring* in *2012 16th international symposium on wearable computers* (2012), 108–109.
17. Sztyler, T. & Stuckenschmidt, H. *On-body localization of wearable devices: An investigation of position-aware activity recognition* in *2016 IEEE International Conference on Pervasive Computing and Communications (PerCom)* (2016), 1–9.
18. Willetts, M., Hollowell, S., Aslett, L., Holmes, C. & Doherty, A. Statistical machine learning of sleep and physical activity phenotypes from sensor data in 96,220 UK Biobank participants. *Scientific reports* **8,** 1–10 (2018).
